# Towards Precision Medicine in Clinical Practice: Alinity C vs. UHPLC-MS/MS in Plasma Aripiprazole Determination

**DOI:** 10.3390/pharmaceutics16010104

**Published:** 2024-01-12

**Authors:** Francisco José Toja-Camba, Enrique Bandín-Vilar, Gonzalo Hermelo-Vidal, Carolina Feitosa-Medeiros, Antonio Cañizo-Outeiriño, Ana Castro-Balado, Iria Varela-Rey, Irene Zarra-Ferro, Anxo Fernández-Ferreiro, Cristina Mondelo-García

**Affiliations:** 1 Pharmacy Department, University Clinical Hospital of Santiago de Compostela (SERGAS), 15706 Santiago de Compostela, Spain; kikotoja@gmail.com (F.J.T.-C.); enriquebandinvilar@gmail.com (E.B.-V.); ana.castro.balado@gmail.com (A.C.-B.); iriavarela13@gmail.com (I.V.-R.); irene.zarra.ferro@sergas.es (I.Z.-F.); 2 Clinical Pharmacology Group, Health Research Institute of Santiago de Compostela (IDIS), 15706 Santiago de Compostela, Spain; zalohermelo@gmail.com (G.H.-V.); carolinafeimed@gmail.com (C.F.-M.); antonio.canizo@gmail.com (A.C.-O.); 3 Faculty of Pharmacy, University of Santiago de Compostela, 15782 Santiago de Compostela, Spain

**Keywords:** aripiprazole, UHPLC-MS/MS, Alinity C, TDM, monitoring

## Abstract

Therapeutic drug monitoring improves the benefit–risk balance of antipsychotic therapy. Ultra-high-performance liquid chromatography–tandem mass spectrometry (UHPLC-MS/MS) is considered the gold-standard method for measuring plasma drug concentrations; however, the Alinity C system has emerged as a promising alternative. This is the first study aimed at comparing UHPLC-MS/MS versus Alinity C in measuring plasma concentrations of aripiprazole and dehydroaripiprazole. A total of 86 plasma samples were analyzed. The active moiety of aripiprazole was measured in 60 samples using both systems and 26 samples were analyzed twice using Alinity C with an intermediate period of 6 months to assess its reproducibility. Spearman’s correlation revealed a good association between the two assays (rs = 0.96) and no significance differences were found by McNemar’s test when classifying samples between infra-, supra- and therapeutic ranges. Passing–Bablock regression showed a good correlation among methods (rs = 0.93) and a slope of 1.12 indicating a slight tendency of Alinity C to measure higher values than UHPLC-MS/MS. In addition, a good intra-method correlation across the two sequential analyses with Alinity C was obtained (rs = 0.99). Nonetheless, clinical decisions could be different in 15% of the cases depending on the chosen method. No differences were found in active moiety determination by Alinity C depending on the concentration of aripiprazole and dehydroaripiprazole of the samples.

## 1. Introduction

Mental health disorders, such as schizophrenia and bipolar disorder, pose a significant public health challenge due to their clinical complexity and substantial impact on patients’ quality of life [1,2]. The effective management of these conditions often involves the use of antipsychotic medications, among which aripiprazole, a second-generation drug, has proven efficacy in treating psychotic and affective symptoms.

Antipsychotic drugs are often far less effective than expected. Treatment resistance requires dose optimization based on a trial-and-error approach because the therapeutic response cannot currently be predicted. Together with serious and frequent adverse events, these are the main reasons leading to treatment discontinuation.

In the clinical setting, changes to the original prescribed dosage shown in the summary of product characteristics (SmPC) are frequently required to achieve an adequate clinical response while minimizing adverse effects. It is a huge challenge for physicians to predict the optimal dose in the absence of objective data such as plasma drug concentrations, due to excessive inter-individual variability. Therefore, this trial-and-error procedure may delay the achievement of clinical goals and even lead to the occurrence of relapse or adverse events, which are closely related to poor prognosis and poor treatment compliance.

Aripiprazole was the first antipsychotic to have a partial agonist role at D2 receptors. This property, plus the D3 partial agonist and 5-HT1A full agonism properties, and the 5-HT2A receptor antagonism, translates into a decrease in the negative, positive and cognitive symptoms of schizophrenia, minimizing the risk of adverse effects in comparison to other atypical antipsychotics [3]. It is metabolized through cytochrome CYP2D6 and CYP3A4 to its main metabolite, dehydroaripiprazole. The therapeutic reference range trough values were reported by the the TDM task force of the Arbeitsgemeinschaft für Neuropsychopharmakologie und Pharmakopsychiatrie (AGNP) between 100 and 350 ng/mL for aripiprazole and 150 and 500 ng/mL for the active moiety (aripiprazole plus dehydroaripiprazole) [4]. Recently, new reference ranges were proposed of 120–270 ng/mL and 180–380 ng/mL, respectively, for aripiprazole and its active moiety by Hart et al. [5].

In recent years, an increasing body of literature has emphasized the significance of personalized medicine in psychiatric care, highlighting the importance of tailoring treatments based on individual patient profiles [6,7,8]. The advent of precision psychiatry has underscored the need for robust analytical methods capable of delivering accurate and reliable data for informed decision-making in the clinical setting [9,10]. In this regard, achieving optimal therapeutic outcomes of aripiprazole requires the precise monitoring of plasma concentrations. In this sense, accurate quantification of aripiprazole is crucial for tailoring treatment strategies to individual patient needs, minimizing adverse effects and optimizing therapeutic efficacy [11,12]. Liquid chromatographic methods for the analysis of aripiprazole are widely described in the literature: there are LC-MS/MS [13,14,15,16], HPLC-UV/DAD [17,18,19] or GC-MS [20]-based methods.

Ultra-high-performance liquid chromatography–tandem mass spectrometry (UHPLC-MS/MS) has emerged as a powerful analytical tool, offering a high sensitivity and selectivity for the quantification of compounds in biological samples. Its ability to discriminate between aripiprazole and its metabolite through mass spectrometry enables precise and specific detection, addressing the demand for accurate therapeutic drug monitoring in psychiatric patients. As the field of psychopharmacology advances, the need for analytical methods that can keep pace with the evolving landscape of psychiatric medications increases. The UHPLC-MS/MS methodology is considered the gold standard in pharmacokinetic studies, providing unparalleled sensitivity and specificity [21]. Its ability to quantify analytes at lower concentrations, coupled with the ability to distinguish between closely related compounds, positions it as an ideal candidate for the analysis of aripiprazole and its metabolite in complex biological matrices. However, sample processing prior to analysis is completely manual, which introduces potential variability, and it is very time consuming.

Conversely, the Alinity C system (Abbot Laboratories), which uses potentiometry and photometry, is a promising alternative, providing advantages in terms of automation and user-friendliness, potentially facilitating implementation in clinical settings. Automation is a hallmark of the Alinity C system, potentially reducing the analytical variability associated with manual sample handling. This feature aligns with the growing demand for high-throughput analytical methods in clinical laboratories, where efficiency and reproducibility are paramount.

This is the first study that compares two analytical methods for the plasma determination of aripiprazole: UHPLC-MS/MS and the Alinity C system, with a focus on sensitivity, precision and accuracy. Furthermore, this study is the pioneering effort to assess the impact of sample freezing on the reproducibility of the analysis using Alinity C, as well as the stability of plasma samples when subjected to this technique. Understanding the comparative advantages and limitations of these methods is mandatory in order to advance therapeutic monitoring practices, ultimately optimizing treatment outcomes for individuals grappling with mental health disorders.

## 2. Materials and Methods

A retrospective study is proposed, where plasma samples of patients treated with aripiprazole monohydrate were selected. The study and data collection strictly adhered to the principles of the Declaration of Helsinki. Ethical approval was given by the local Institutional Review Board and the autonomous region of Galicia (2021/285).

### 2.1. UHPLC-MS/MS

Concentrations of aripiprazole and dehydroaripiprazole in plasma were measured by a validated UHPLC-MS/MS method on a Xevo TQD^®^ triple quadrupole mass spectrometer (Waters, Milford, MA, USA). Aripiprazole-d8 (deuterated aripiprazole) was used as the internal standard. Aripiprazole, dehydroaripiprazole and aripiprazole-d8 detection were monitored at the following transition pairs m/z 448.2 → 285.2, m/z 446.04 → 285.02 and m/z 456.3 → 293.07, respectively.

For this purpose, 850 µL of plasma with 100 µL of aripiprazole and dehydroaripiprazole at a concentration of 0.25 mg/L and 50 µL of aripiprazole-d8 at a concentration of 10 mg/L are added in an Eppendorf and shaken by vortex-mixing for 1 min. Four samples were extracted from this solution, each consisting of 200 µL of the combined solution and 600 µL of acetonitrile. Subsequently, they were vortex-mixed for 1 min and centrifuged (3500 rpm 10 min 4 °C) and 500 µL of the resulting supernatant are extracted. Then, the samples were evaporated to dryness and finally dissolved in 500 µL of a solution composed of water and acetonitrile in a ratio of 80:20.

A linear calibration curve was obtained over a concentration range of 25–1000 ng/mL (R2 = 0.998). The detection and quantification limits for both aripiprazole and dehydroaripiprazole were 10 and 25 ng/mL, respectively. An ACQUITY UHPLC BEH C18 column (2.1 × 50.0 mm, 1.7 μm) was used at a temperature of 40 °C. The sample manager was set to 4 °C, the flow rate to 0.6 mL/min and the injected volume of sample to 5 μL. The obtained data were processed using MassLynx^®^ software (Version 4.20.0001).

### 2.2. Alinity C

Alinity C is based on photometric and potentiometric detection technologies for the quantitative determination of analytes in human serum, plasma, urine, cerebrospinal fluid, hemolysate and whole blood. Alinity C assay for determination of the active moiety of aripiprazole is based on photometric detection, using a tungsten lamp, a diffraction grating and a silicon photodiode detector. The test was performed at a wavelength of 604 nm. The MyCare Psychiatry Kit for total aripiprazole (Saladax Biomedical, Bethlehem, PA, USA) was used to determine the concentration of active moiety. It is based on competition between drug and drug-conjugates for binding to drug specific antibodies covalently bound to nanoparticles. The extent of particle aggregation can be followed spectrophotometrically on clinical chemistry analyzers as Alinity C. Cross-reactivity was tested by the kit manufacturer using more than 150 drugs as well as the presence of interfering substances such as rheumatoid factor, lipids and hemolyzed blood. The lower limit of quantification of the kit is 45 ng/mL and the upper limit of quantification is 1000 ng/mL.

Since the determination by Alinity C only provides values of active moiety (aripiprazole plus dehydroaripiprazole), the sum of drug and active metabolite found by UHPLC-MS/MS has been used for the comparison between both methods.

A total of 86 plasma samples, corresponding to trough concentrations from 86 patients, were collected. Samples were stored at −80 °C until analysis.

On the one hand, 60 samples were analyzed by both UHPLC-MS/MS and Alinity C. Due to the clinical repercussions of possible variations in the use of one method or another, the samples have been stratified according to the consensus therapeutic range into infra-therapeutic (<150 ng/mL), therapeutic (150–500 ng/mL) and supra-therapeutic (>500 ng/mL). Furthermore, it was studied whether there were differences between the two methods when the aripiprazole/dehydroariprazole (ARI/DHA) ratios were different, and thus whether the amount of each analyte individually affected the determination by Alinity C. For this purpose, the proportion between the concentration measured by the two methods (UHPLC-MSMS/Alinity C) was classified into 5 different groups according to the ARI/DHA ratio (1–2, 2–3, 3–4, 4–5, 5–6).

On the other hand, 26 samples previously analyzed by Alinity C and stored at −80 °C were selected and reanalyzed by the same method 6 months later to determine the reproducibility of the method and the stability of the plasma samples.

### 2.3. Statistics

Normality was tested using the Shapiro–Wilk test, and correlations were assessed using the Spearman rank correlation coefficient (rs). The Wilcoxon rank sum test was used to determine differences between drug levels. Bland–Altman analysis was performed to assess the agreement between the two quantitative measurements. A Bland–Altman plot shows the difference (y-axis) between these two measurements. The mean (x-axis) represents the average difference between two measurements, and the limits of agreement are expressed as the mean difference plus or minus 2 standard deviations of the difference. Additionally, Passing–Bablock regression was performed to estimate the best-fit line by comparing observed ranks between concentrations measured by UHPLC-MS/MS and Alinity C and measurements from two consecutive Alinity C analyses.

Agreement between classification of UHPLC-MS/MS and Alinity C into three different therapeutic range groups was determined using weighted Cohen kappa (κ), and differences in classification for each group were assessed using McNemar’s test. To assess if the presence of higher concentrations of aripiprazole or its active metabolite dehydroaripiprazole affects the active moiety determined by Alinity C system, an ANOVA test was performed to determine differences between group means.

## 3. Results

### 3.1. UHPLC-MS/MS vs. Alinity C

Aripiprazole active moiety concentrations were measured by both methods. The median values were 230.6 ng/mL (IQR: 166.2–418.8) for the UHPLC-MS/MS assay and 239 ng/mL (IQR: 167.8–456.5) for the Alinity C assay. The UHPLC-MS/MS method yielded a mean concentration for active moiety of 275.3 ng/mL, with a standard deviation of 140.2. In comparison, the Alinity C method exhibited a slightly higher mean concentration of 299.9 ng/mL, with a standard deviation of 162.5 (Table 1). The Spearman’s rank correlation coefficient showed a good correlation in active moiety values between the two tests (rs = 0.96).

#### 3.1.1. Bland–Altman and Regression Analysis

**Figure 1:**
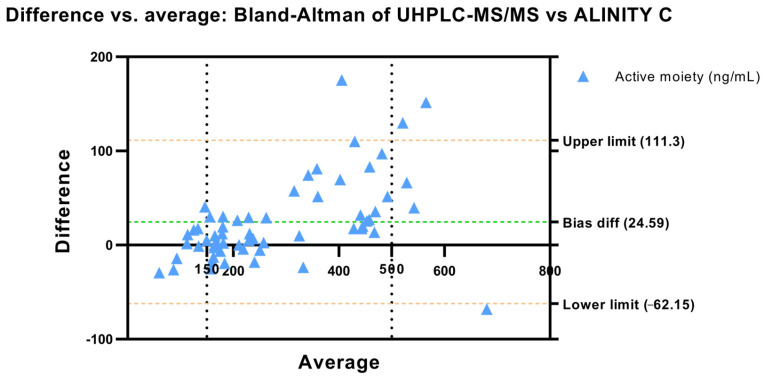
Bland–Altman’s plot. Difference in active moiety concentrations vs. the average (ng/mL) between UHPLC-MS/MS and Alinity C. The dashed green line represents the bias and dashed orange lines represent the limit of agreement.

**Figure 2:**
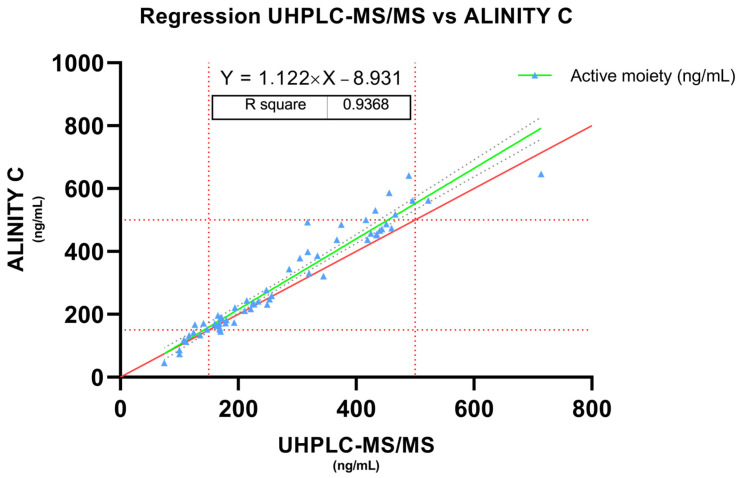
Passing–Bablock. Regression of measured concentrations of the active moiety between the UHPLC-MS/MS and Alinity C system. Red line represents the identity line. Grey dashed lines are the 95% confidence bounds. Dashed red lines represents aripiprazole therapeutic range (active moiety).

In the present study, we conducted a Bland–Altman analysis to assess the agreement between two measurement methods (Figure 1). The mean difference, or bias, between the methods was found to be 5.560. The standard deviation of the bias was 14.28. The 95% limits of agreement, calculated using the Bland–Altman method, were determined to be −22.43 and 33.55, establishing an interval within which it is expected that 95% of the differences between the methods will fall. These findings were supported by the regression that estimated the line of best fit between both methods (Figure 2). Revealing a slope of 1.122, an intercept of −8.931 and a high correlation coefficient (r) of 0.936. The *p*-value (<0.0001), which is significantly below the conventional threshold of 0.05, underscores the statistical significance of this correlation. Furthermore, the F-test, with an F-statistic of 860.4 and a *p*-value < 0.0001, emphasizes the statistical significance of the slope, confirming a significant deviation from zero.

#### 3.1.2. Stratification in Ranges

The concentrations determined by both methods were classified according to the sub- (<150 ng/mL), supra (>500 ng/mL) and therapeutic range (>500 ng/mL). Cohen’s weighted kappa showed a substantial agreement between the measurements of both methods (K = 0.643 ± 0.105). The qualitative agreement between the two analytical methods was up to 84.99% of the measured samples. Considering this percentage in each range, the concordance of results was 8/9 (88.88%) samples for the sub-therapeutic range, 41/44 (93.18%) for the therapeutic range and 2/7 (28.5%) for the supratherapeutic range. The therapeutic strategy decision could have been different in nine samples (15%) (Table 2). To complete the comparison, McNemar’s test was performed to determine whether the classification was discordant between the subtherapeutic and therapeutic range and between the therapeutic and supratherapeutic range. No significant differences (*p* = 0.625 and *p* = 0.0625) were found between the classification between these groups by both methods.

Since the Alinity C system does not provide concentrations of aripiprazole and dehydroaripipiprazole singularly, but only reports the active moiety, a stratification according to the ARI/DHA ratio obtained by UHPLC-MS/MS was conducted to check the influence of both analytes (ARI and DHA) concentrations over Alinity C performance and to verify that the measurements were not dependent on the concentration of a particular analyte. For this purpose, an ANOVA test was performed which revealed no significant differences (*p* = 0.587) between the means of the differences between UHPLC-MS/MS and Alinity C between the five groups as a function of the ARI/DHA ratio.

### 3.2. Alinity C Reanalysis

A total of 26 samples were analyzed twice 6 months apart by Alinity C to determine the consistency of the method. During this period, samples were stored at −80 °C. Descriptive analyses of the results are available in Table 1.

**Figure 3:**
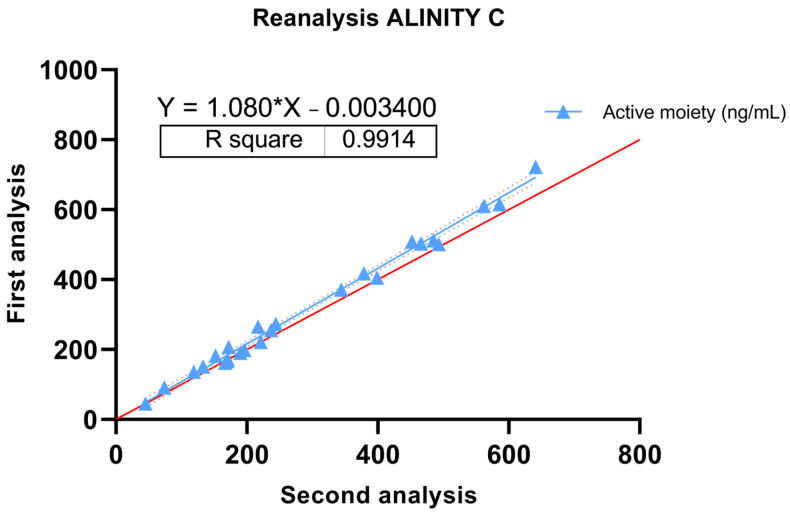
Passing–Bablock. Regression of measured concentrations of the active moiety between the first and second analysis by the Alinity C system. Red line represents the identity line. Grey dashed lines are the 95% confidence bounds.

Linear regression revealed a slope of 1.080, a y-intercept of −0.003400 and an x-intercept of 0.003148. The model demonstrates an exceptional goodness of fit, with an R-square value of 0.9914, The F-test, with an F-statistic of 2759 and a *p*-value less than 0.0001, underscores the statistical significance of the slope, confirming a significant deviation from zero (Figure 3).

## 4. Discussion

The landscape of psychiatric pharmacotherapy is continuously evolving, requiring analytical methods that can provide accurate and reliable data for personalized treatment approaches. As far as we know, this study stands out as the initial attempt to compare these two methods in determining aripiprazole, a drug that has accumulated substantial evidence supporting the need for monitoring its plasma concentrations.

In the field of psychiatry research, the choice between analytical methods extends beyond technical considerations to encompass practical aspects, such as cost, ease of use and scalability [22]. The Alinity C system, with its potential for automation and simplified workflows, may present advantages in resource-limited clinical settings where short turnaround times and cost-effectiveness are crucial considerations. Conversely, the UHPLC-MS/MS method, while more complex, offers unparalleled sensitivity and specificity, making it indispensable for research settings and situations where the highest level of analytical precision is required.

The Bland–Altman approach provides a comprehensive insight into the agreement between measurements, offering a nuanced understanding of the potential discrepancies between the two methods under consideration. Bland–Altman analysis was conducted to rigorously evaluate the agreement between two distinct measurement methods. The mean difference, indicative of bias, was calculated at 5.56, which indicates a systematic tendency towards higher values in Alinity C analysis. In parallel, the regression analysis confirms these results showing a slope of 1.122, suggesting a systematic tendency for the Alinity C system to yield slightly higher values than the UHPLC-MS/MS, especially at high concentration values.

The high correlation coefficient (r) of 0.936 further underscores the strength and direction of the relationship between the two methods. This implies that a substantial proportion of the variability in one method can be explained by the variability in the other, reinforcing the notion of a robust association.

The stratification of concentrations into sub-therapeutic, therapeutic and supratherapeutic ranges showed substantial agreement between the two methods, with a Cohen’s weighted kappa of 0.643 ± 0.105 [23]. The qualitative agreement was high across the ranges, particularly in the therapeutic range (93.18%) and sub-therapeutic range (88.88%). The McNemar test indicated no significant differences in classification between the sub-therapeutic and therapeutic or therapeutic and supratherapeutic ranges, emphasizing the reliability of both methods in guiding aripiprazole dosing towards the achievement of the commonly targeted therapeutic ranges.

A total of 3/11 (27.7%) patients with sub-therapeutic ranges measured by UHPLC-MS/MS were classified as therapeutic by Alinity C, and 5/47 (10.6%) in a therapeutic range by UHPLC-MS/MS were classified as supra-therapeutic by Alinity C. Taking into account all the discrepancies, the therapeutic decision to be taken would be different on nine occasions (15%) according to the method used. This leads us to realize that, in case of doubt and especially at high plasma concentrations, it is always necessary to confirm with a reference method and to be cautious before making clinical decisions.

In addition, to determine whether the presence of a higher concentration of aripiprazole or its active metabolite dehydroaripiprazole influences the active moiety determined by Alinity C, we compared the ratio between the result of the analysis by Alinity C and UHPLC-MS/MS and grouped them according to the ARI/DHA ratio, proving that Alinity C is a robust method regardless of the concentration of both analytes. This is important to know, since the ratio between ARI/DHA does not remain constant over the time of administration or between different patients, especially in those with metabolizing phenotypes for CYP2D6 different from the normal phenotype [8].

UHPLC-MS/MS, while considered the gold standard in pharmacokinetic studies, has manual sample processing, introducing potential variability and time-consuming steps [24]. Its high sensitivity and specificity, especially in distinguishing closely related compounds, makes it valuable for research purposes [25,26]. The Alinity C system, on the other hand, offers automation, reducing the analytical variability associated with manual sample handling. Its user-friendliness makes it suitable for routine clinical use. However, it is essential to note that it provides values only of the active moiety and not of each separate compound.

When it comes to clinical implementation, the choice between UHPLC-MS/MS and the Alinity C system should consider the specific needs of the clinical setting. UHPLC-MS/MS remains valuable for research and situations requiring the highest sensitivity and specificity. However, the Alinity C system’s automation and user-friendliness makes it an attractive option for routine therapeutic drug monitoring, offering efficiency without compromising accuracy [27].

On the other hand, our study also assessed the consistency of the Alinity C method and the stability of plasma samples through the reanalysis of 26 samples within a 6-month interval. Linear regression analysis of the reanalyzed samples demonstrated a slope of 1.080, indicating a minimal increase in concentration over time. This 8% increase at six months is within the ±15% variation allowed [28]. The exceptionally high R-square value of 0.991 suggests a robust fit of the linear regression model, highlighting the consistency of the Alinity C method over the storage period in the aforementioned conservation conditions. The F-test with a significant F-statistic of 2759 and a *p*-value less than 0.001 further confirmed the statistical significance of the slope, reinforcing the reliability of the reanalysis results. These findings suggest that the Alinity C method maintains consistency and accuracy even after storing samples at −80 °C for six months. In this sense, Jansen et al. published the only other study which evaluates by this same technique the stability of samples after freezing, with similar results [29].

While the study provides evidence into the comparability and consistency of the UHPLC-MS/MS and Alinity C methods, certain limitations should be acknowledged. The sample size, although sufficient for the intended analysis, might limit the generalizability of the findings to a broader population. Additionally, the discordance observed in the supratherapeutic range warrants further investigation. Future studies could explore factors contributing to this discrepancy, such as the presence of interfering substances or method-specific limitations in accurately quantifying higher concentrations.

The advent of precision psychiatry underscores the importance of analytical methods capable of delivering accurate and reliable data for informed decision-making. As the field of psychopharmacology continues to advance, the ongoing evaluation of analytical methodologies is essential to ensure that clinical practices align with the evolving landscape of psychiatric therapies. This study contributes to this ongoing effort by providing evidence for the suitability of the Alinity C system as a practical and reliable tool for the therapeutic monitoring of aripiprazole in these patients. These findings contribute to the ongoing dialogue in refining analytical methods for therapeutic drug monitoring, ultimately benefiting clinicians and patients in ensuring the accurate and consistent assessment of antipsychotic medication concentrations in plasma.

The findings support the integration of the Alinity C system into clinical laboratories, emphasizing its potential to enhance efficiency and reproducibility in therapeutic monitoring practices. However, it is crucial to acknowledge the limitations of each method and carefully consider the specific requirements of individual clinical scenarios.

## 5. Conclusions

This study provides valuable insights into the comparative analysis of UHPLC-MS/MS and the Alinity C system for the plasma determination of aripiprazole and reproducibility after freezing samples. Both methods demonstrated a high precision and substantial agreement, particularly in the therapeutic range. These results affirm the incorporation of the Alinity C system into clinical laboratories, highlighting its capacity to improve effectiveness and consistency in therapeutic drug monitoring. However, it is essential to recognize the constraints of each approach and meticulously assess the unique needs of different clinical situations.

Although systematic upward method drift must be taken into account, the Alinity C system with its automation and ease-of-use features presents a promising alternative for routine clinical use, addressing the need for high-throughput analytical methods.

## Figures and Tables

**Table 1:** Descriptive statistics of both assays.

	UHPLC-MS/MS Dehydroaripiprazole (ng/mL)	UHPLC-MS/MS Aripiprazole (ng/mL)	UHPLC-MS/MS Active Moiety (ng/mL)	Alinity C Active Moiety (ng/mL)	Ratio ARI/DHA UHPLC-MS/MS
Samples	60	60	60	60	60
Minimum	21.01	53.40	74.40	45.00	1.32
Maximum	185.30	551.90	714.10	646.00	5.23
25% Percentile	49.81	114.40	166.20	167.80	169.30
Median	66.01	162.50	230.60	239.00	229.00
75% Percentile	99.32	289.20	418.80	456.50	455.50
Mean	77.84	197.40	275.30	299.90	2.54
Std. Deviation	36.43	110.60	140.20	162.50	0.86
Std. Error of Mean	4.70	14.28	18.10	20.97	0.11
Coefficient of variation	46.81%	56.02%	50.93%	54.18%	34.08%

**Table 2:** Stratification of samples between the three possible ranges according to UHPLC-MS/MS and Alinity C active moiety measured concentrations.

		Number of Samples (%)				Statistics		
		Alinity C				Cohen’s Weighted Kappa		
Active Moiety		<150 ng/mL	150–500 ng/mL	>500 ng/mL	Total	K	SE	CI 95%
**UHPLC-MS/MS**	**<150 ng/mL**	8 (13.33)	3 (5)	0	11 (18.33)	0.643	0.105	0.437–0.849
	**150–500 ng/mL**	1 (1.67)	41 (68.33)	5 (8.33)	47 (78.33)	**McNemar test**		
	**>500 ng/mL**	0	0	2 (3.33)	2 (3.33)	**(<150 ng/mL)-** **(150–500 ng/mL)**	**(150–500 ng/mL)-(>500 ng/mL)**	
	**Total**	9 (15)	44 (73.33)	7 (11.67)	60 (100)	*p*-value = 0.625	*p*-value = 0.0625	

## Data Availability

Data are contained within the article.
